# Construction and validation of a SASP‐related prognostic signature in patients with acute myeloid leukaemia

**DOI:** 10.1111/jcmm.70017

**Published:** 2024-08-19

**Authors:** Ming‐Feng Li, Dong‐Hui Zhang, Li‐Si Wang, Cai‐Feng Yue, Li‐Juan Pang, Yun‐Miao Guo, Zhi‐Gang Yang

**Affiliations:** ^1^ Zhanjiang Institute of Clinical Medicine, Central People's Hospital of Zhanjiang, Guangdong Medical University Zhanjiang Central Hospital Zhanjiang China; ^2^ Department of Laboratory Medicine, Central People's Hospital of Zhanjiang Guangdong Medical University Zhanjiang Central Hospital Zhanjiang China; ^3^ Department of Pathology, Central People's Hospital of Zhanjiang Guangdong Medical University Zhanjiang Central Hospital Zhanjiang China; ^4^ Department of Hematology, Central People's Hospital of Zhanjiang Guangdong Medical University Zhanjiang Central Hospital Zhanjiang China

**Keywords:** acute myeloid leukaemia (AML), immune infiltration, prognosis, risk signature, senescence‐associated secretory phenotype (SASP)

## Abstract

Acute myeloid leukaemia (AML) is a common and highly aggressive haematological malignancy in adults. Senescence‐associated secretory phenotype (SASP) plays important roles in tumorigenesis and progression of tumour. However, the prognostic value of SASP in patients with AML has not been clarified. The present study aims to explore the prognostic value of SASP and develop a prognostic risk signature for AML. The RNA‐sequencing data was collected from the TCGA, GTEx and TARGET databases. Subsequently, differentially expressed gene analysis, univariate Cox regression and LASSO regression were applied to identified prognostic SASP‐related genes and construct a prognostic risk‐scoring model. The risk score of each patient were calculated and patients were divided into high‐ or low‐risk groups by the median risk score. This novel prognostic signature included 11 genes: *G6PD*, *CDK4*, *RPS6KA1*, *UBC*, *H2BC12*, *KIR2DL4*, *HSF1*, *IFIT3*, *PIM1*, *RUNX3* and *TRIM21*. The patients with AML in the high‐risk group had shorter OS, demonstrating that the risk score acted as a prognostic predictor, which was validated in the TAGET‐AML dataset. Univariate and multivariate analysis revealed the risk score was an independent prognostic factor in patients with AML. Furthermore, the present study revealed that the risk score was associated with immune landscape, immune checkpoint gene expression and chemotherapeutic efficacy. In the present study, we constructed and validated a unique SASP‐related prognostic model to assess therapeutic effect and prognosis in patients with AML, which might contribute to understanding the role of SASP in AML and guiding the treatment for AML.

## 1 INTRODUCTION

Acute myeloid leukaemia (AML) is the most common acute blood malignancy in adults and is caused by proliferative and differential abnormalities of myeloid progenitor cells in the bone marrow (BM) microenvironment.^1^ AML has a high mortality rate due to rapid progression, high heterogeneity and frequent mutation. Moreover, AML is prevalent in elders, with a median age of 69 at diagnosis. Despite substantial therapeutic development making about 35% ~ 40% of young patients cured, the outcome of the older patients with AML remains undesirable.^2^ It is essential to identify novel biomarkers for risk stratification and accurate prognosis in patients with AML, especially in elders.

Senescent cells secrete numerous proteases, inflammatory cytokines and growth factors for recruitment of immune cells including macrophages, natural killer (NK) cells, neutrophils and T lymphocytes to eliminate senescent cell, which are termed as the senescence‐associated secretory phenotype (SASP).^3^ However, accumulating results indicate that SASP could promote the cell proliferation,^4^, ^5^ metastasis and tumour progression.^6^, ^7^ SASP creates an immunosuppressive microenvironment to facilitate tumour growth and immune escape. Furthermore, SASP has significant impacts on the effect of tumour chemotherapy by causing adverse reactions such as myelosuppression, heart failure and tumour recurrence.^8^, ^9^ Thus, elucidating the prognostic significance of SASP can provide insight into its function in AML and guide the treatment.

In the present work, we sought to explore the predictive value of SASP‐related genes by constructing a prognostic risk‐scoring model based on RNA expression profiles in patients with AML. Subsequently, the performance of this 11 genes model was verified in both of TCGA and TARGET datasets. Furthermore, we interrogated the associations between risk score and response to treatment of AML to assess its potential for predicting the treatment efficiency and prognosis in patients with AML.

## 2 MATERIALS AND METHODS

### 2.1 Data acquisition

The RNA‐seq and clinical data of TCGA, GTEx and TARGET were downloaded from University of California, Santa Cruz (UCSC) Xena Browser.^10^ The different sets of transcript expression data were re‐calculated and normalised by using UCSC TOIL recompute pipeline. A total of 173 AML samples from TCGA cohort and 337 skin samples from GTEx database were recruited in the training set to construct the predictive model. The TARGET cohort of 196 AML samples was utilised as validation set. Subsequently, patients with overall survival (OS) less than 30 days or missing clinical information were excluded for further analyses. Moreover, the list of SASP‐related genes was acquired by searching in the GeneCards database.^11^

### 2.2 Differentially expression analysis and univariate Cox regression analysis

The R package DESeq2^12^ was adopted to screen the differentially expressed gene (DEG) with a threshold of |log_2_ (Fold Change)| >1 and *p* value <0.05, following by visualization with R package ComplexHeatmap.^13^ Meanwhile, univariate Cox regression analysis was used to identified the SASP‐related genes associated with length of overall survival time in patients with AML, which were further intersected with differentially expressed SASP‐related genes.

### 2.3 Construction and validation of the prognostic risk‐scoring model

Least Absolute Shrinkage and Selection Operator (LASSO) regression were applied with the 63 candidate SASP‐related genes to remove redundant prognostic genes and establish the prognostic model by using R package glmnet.^14^ Eleven SASP‐related genes were ultimately included to construct the prognosis signature for AML. Based on the coefficient of risk genes, we calculated the risk score for each AML patient according to the formula of *G6PD* × 0.0981 + *CDK4* × 0.0874 + *RPS6KA1* × 0.0956 + *UBC* × 0.2568 + *H2BC12* × 0.0317 + *KIR2DL4* × 0.0668 + *HSF1* × 0.0480 + *IFIT3* × 0.0300 + *PIM1* × 0.0698 + *RUNX3* × 0.0325 + *TRIM21* × 0.0412. The same formula was used in both of training and validation cohorts. Risk score of each patient was calculated and categorised into high‐ and low‐risk groups according to the median risk score. The survival difference of both groups was compared using R packages survival and survminer, and 1‐, 3‐ and 5‐year receiver operator characteristic (ROC) curve analyses were performed by R packages ‘time‐ROC’.^15^

### 2.4 Enrichment of functions and signalling pathways analysis

The DESeq2 package was performed to identify DEG with the same criteria mentioned above in TCGA‐AML patients between the two risk groups. Functional enrichment analysis for the DEG was based on Gene Ontology (GO) and Kyoto Encyclopedia of Genes and Genomes (KEGG) databases using the R package clusterProflier.^16^

### 2.5 Analyses of immune infiltration, immunotherapeutic efficacy and drug sensitivity

Single‐sample gene set enrichment analysis (ssGSEA) in R package GSVA^17^ was used to determine the proportions of 28 immune cell subtypes in each AML patient. Stromal score, immune score, ESTIMATE score and tumour purity were also compared between high‐ and low‐risk groups using the R package estimate.^18^ Drug sensitivity was predicted by using the R package pRRophetic^19^ with the pharmacogenomics data from Genomics of Drug Sensitivity in Cancer (GDSC)^20^ to calculated the half‐maximal inhibitory concentration (IC_50_).

### 2.6 Prognostic analysis and establishment of the predictive nomogram

To further explore the relationships between clinicopathologic characteristics and AML patients' prognosis, we extracted the clinical data including AML risk category, age, gender, race and class from the TCGA and TARGET cohorts. Univariate and multivariate Cox regression analyses were performed to identify the independent prognostic factors, with which a nomogram was constructed by the R package ‘rms’. Subsequently, 1‐, 3‐ and 5‐year calibration curves were applied to discriminate and predict the values of a nomogram.

### 2.7 Statistics

All data analyses and visualizations were carried out in R version 4.0.2. Statistical analysis was carried out using the log‐rank test for univariate Cox regression analysis. Pearson's correlation analysis was performed to evaluate the association between the risk score and immune checkpoints, immune cell infiltration score and characteristic gene expression, respectively. Student's *t* test was performed to assess the statistical significance among variables. *p* < 0.05 was considered as statistically significant unless otherwise specified.

## 3 RESULTS

### 3.1 Identification of differentially expressed SASP‐related genes with prognostic value

**FIGURE 1:**
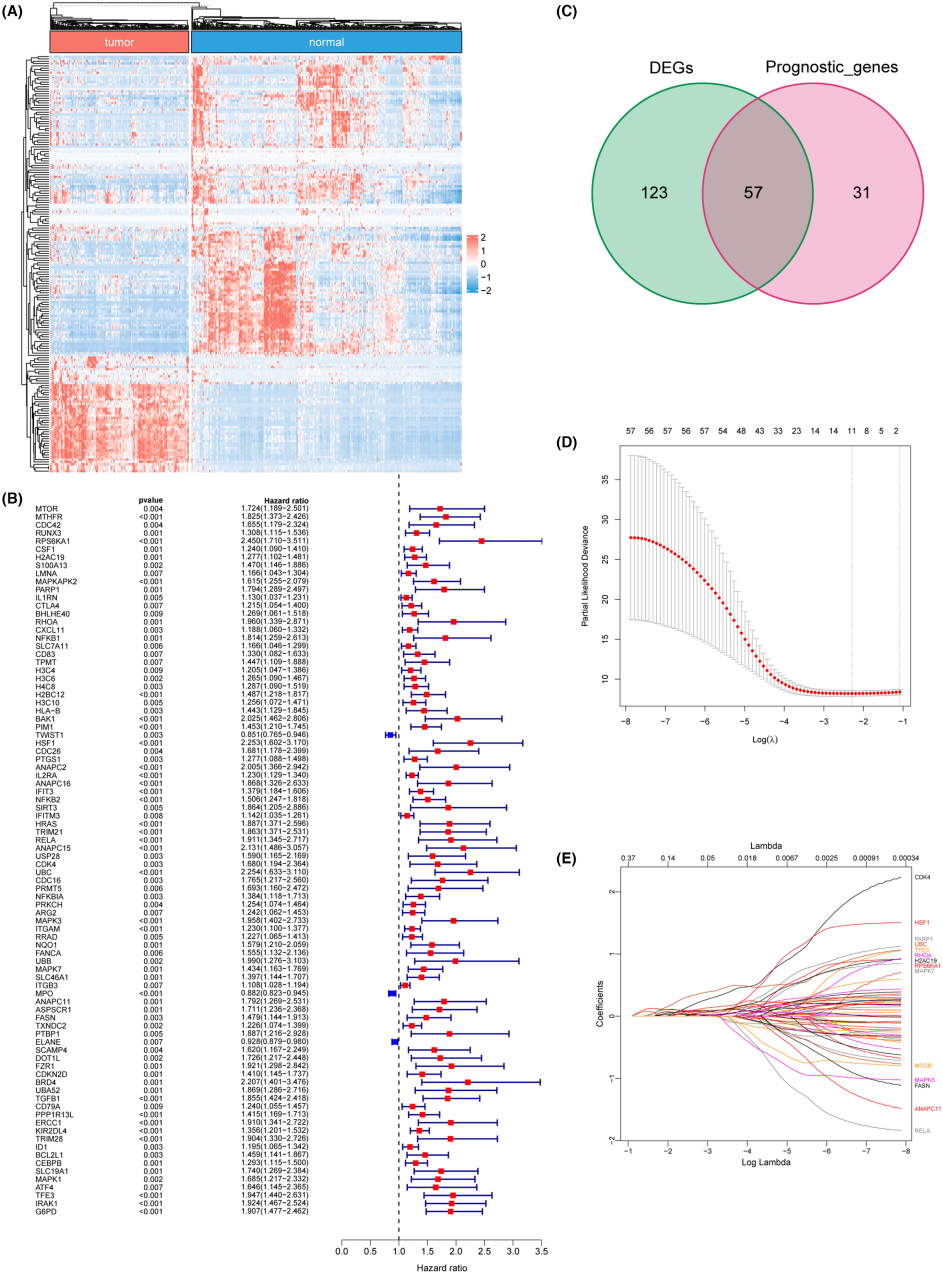
Identification of differential expressed SASP‐related genes with prognostic value. (A) expression of differential expressed SASP‐related genes. (B) Univariate Cox regression analysis showed the hazard ratios of 88 SASP‐related genes correlated with AML prognosis (*p* < 0.05). (C) Intersection of differential expressed SASP‐related genes and prognosis related genes. (D, E) LASSO regression analysis narrowed down the genes to 11.

RNA‐seq of 173 AML samples and 337 normal blood samples from TCGA and GTEx cohorts were downloaded by using UCSC Xena, and 313 SASP‐related genes were extracted from GeneCards, respectively. A total of 134 downregulated and 46 upregulated SASP‐related genes were identified in AML samples compared to normal blood samples, with a criteria of |log_2_ (Fold Change)| >1 and *p* < 0.05 (Figure 1A). Meanwhile, univariate Cox analysis identified 88 SASP‐related genes significantly associated with the OS of AML patients (Figure 1B). Finally, 57 candidate genes overlapped between the gene sets of both DEGs and OS‐associated genes were selected for further analyses (Figure 1C).

### 3.2 Construction of prognostic risk‐scoring model based on SASP‐related genes

**FIGURE 2:**
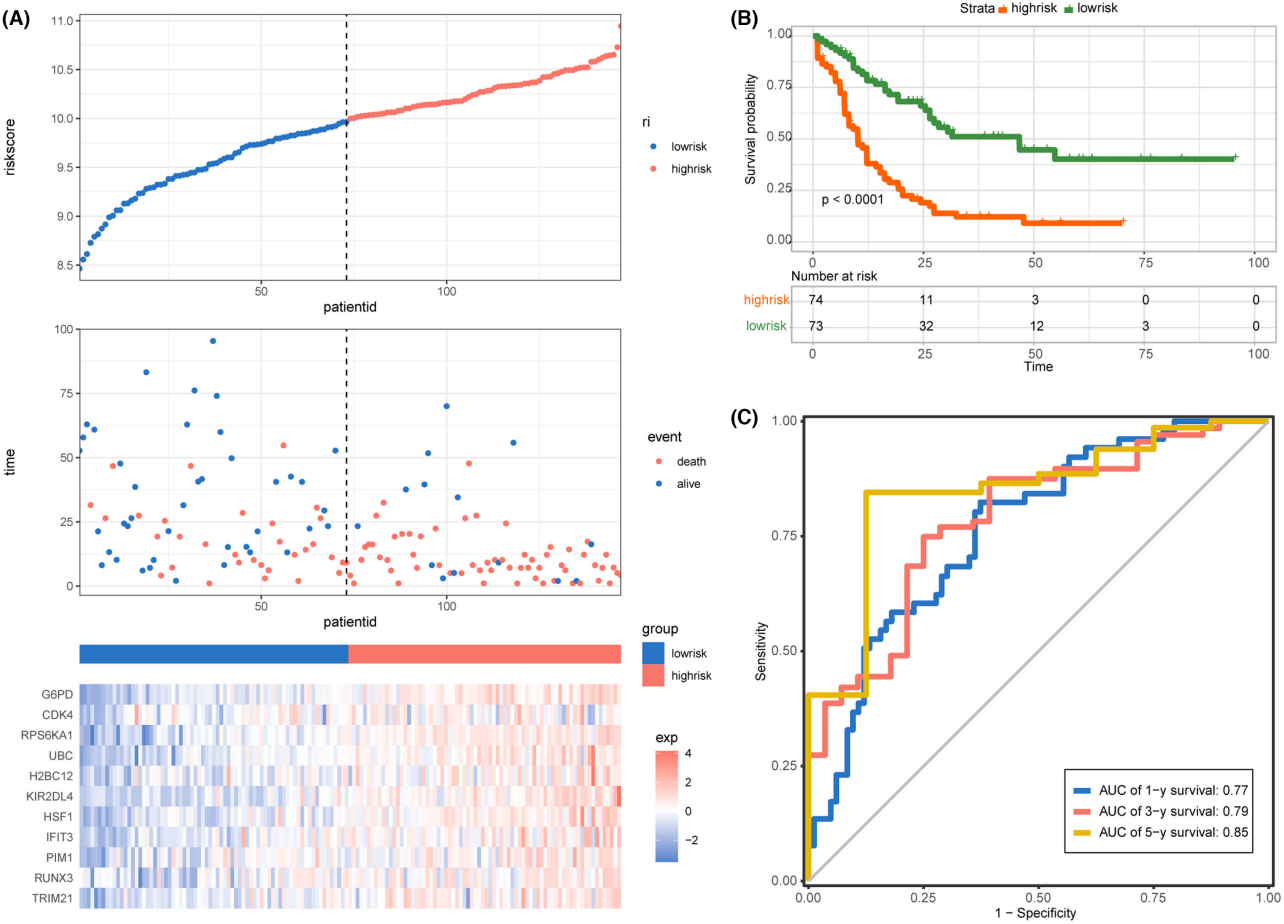
Prognostic analysis of the prognosis model in the training cohort. (A) The distribution of risk score, survival status and expression patterns of each patient. (B) The survival analysis of the two risk groups classified by the signature. (C) ROC curves predicting the 1‐, 3‐, 5‐year OS at 0.77, 0.79, 0.85, respectively.

Lasso regression was employed to construct the prognostic risk‐scoring model and a total of 11 genes including *G6PD*, *CDK4*, *RPS6KA1*, *UBC*, *H2BC12*, *KIR2DL4*, *HSF1*, *IFIT3*, *PIM1*, *RUNX3* and *TRIM21* were ultimately identified, with coefficients of 0.0981, 0.0874, 0.0956, 0.2568, 0.0317, 0.0668, 0.0480, 0.0300, 0.0698, 0.0325 and 0.0412, respectively (Figure 1D,E). Subsequently, the risk scores of each AML patient were calculated in the training dataset. As a result, patients were divided in either the high‐risk group (*n* = 74) or low‐risk group (*n* = 73) using the median of risk scores as cut‐off (Figure 2A). The Kaplan–Meier curve showed that patients in the high‐risk group had shorter OS time than patients in the low‐risk group (Figure 2B). Time‐dependent ROC curve revealed that the AUC of 1‐, 3‐ and 5‐year survival were 0.77, 0.79 and 0.85, respectively (Figure 2C). These results indicated that the novel 11‐gene prognostic model was efficient to predict the prognosis of AML.

### 3.3 Validation of the prognostic signature using TARGET‐AML samples

**FIGURE 3:**
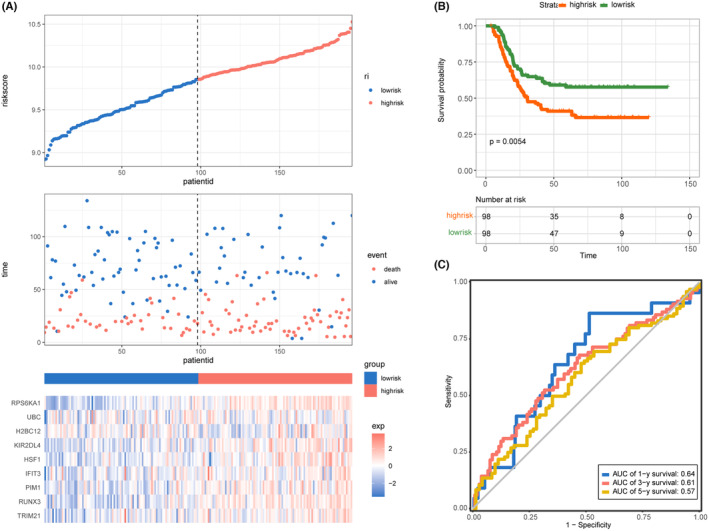
Validation of the signature in the TARGET‐AML cohorts. (A) The distribution of risk score, survival status and expression patterns of each patient. (B) The survival analysis of the two risk groups classified by the signature. (C) ROC curves predicting the 1‐, 3‐, 5‐year OS at 0.64, 0.61, 0.57, respectively.

The risk score of each AML patient in the TARGET cohort was calculated to assign the patients into low‐risk and high‐risk groups (Figure 3A). As expected, patients in the high‐risk group had shorter OS time than patients in the low‐risk group (Figure 3B). Moreover, the AUC of 1‐, 3‐ and 5‐year survival were 0.64, 0.61 and 0.57, respectively (Figure 3C), indicating a table and robust predictive ability of the prognostic model in patients with AML.

### 3.4 Establishment of prognostic nomogram for AML

**FIGURE 4:**
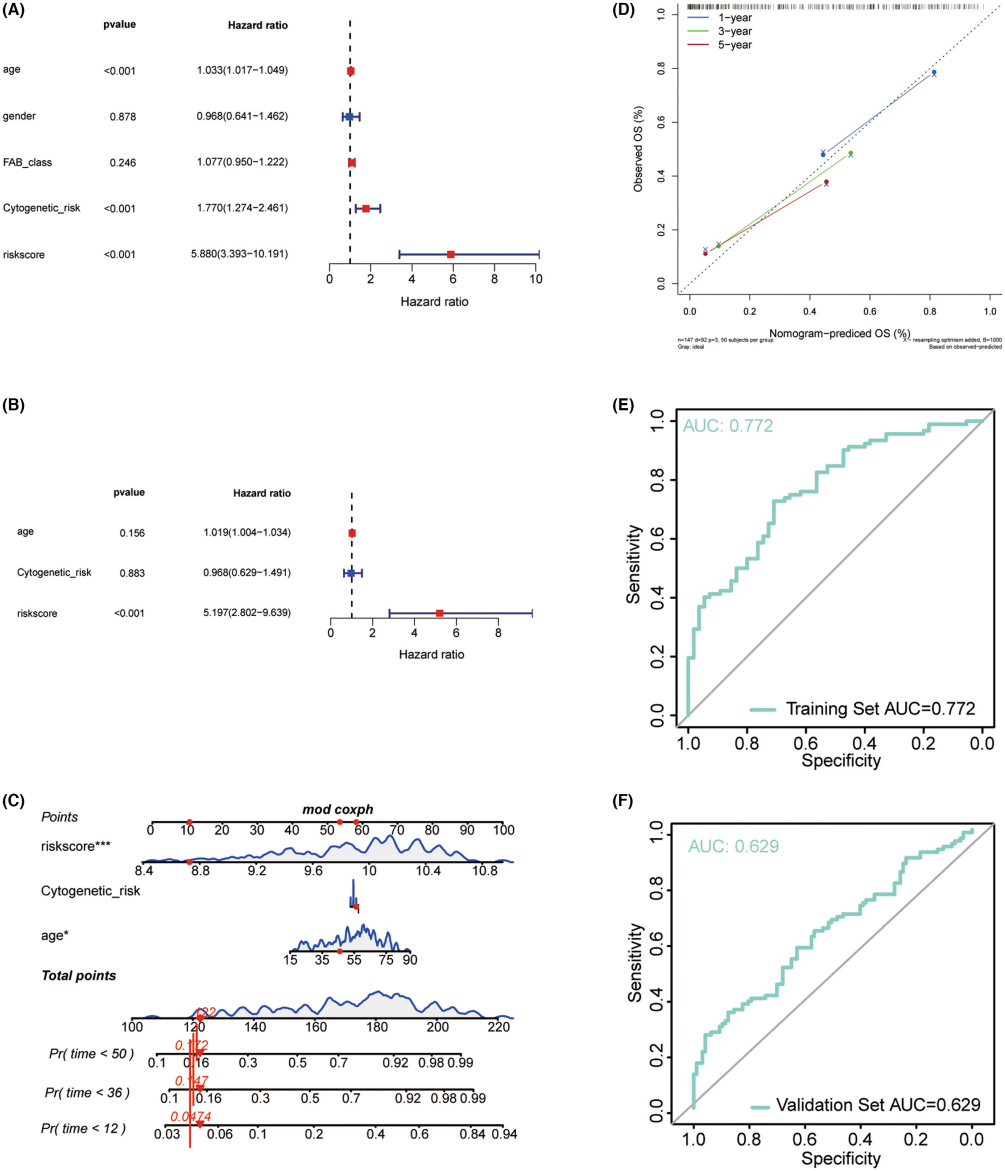
Construction and validation of the nomogram. Univariate (A) and multivariate (B) analyses showed that risk score was an independent prognostic factor in the TCGA‐AML cohort. (C) Nomogram predicting the 1‐, 3‐, 5‐year OS of AML patients. (D) The calibration plot predicting the probability of survival and actual survival rate at 1‐, 3‐, 5‐year OS. (E) ROC curves of nomogram in the TCGA‐AML cohort. (F) ROC curves of nomogram in the TARGET‐AML cohort.

Univariate COX analyses revealed that age (HR = 1.033, *p* < 0.001), cytogenetic risk (HR = 1.770, *p* < 0.001) and risk score (HR = 5.880, 95% CI = 3.393 ~ 10.191, *p* < 0.001) were significantly associated with the overall survival outcome of patients with AML (Figure 4A). Moreover, the result of multivariate COX analysis demonstrated that risk score (HR = 5.197, *p* < 0.001) was the only independent prognostic factor of AML (Figure 4B). Based on the selected variables, a nomogram was established to predict the survival probability of the AML patients in 1, 3 and 5 years (Figure 4C). The results of calibration curve analysis showed that the forecasting curves of 1‐, 3‐ and 5‐years OS was substantially consistent with those of the observed OS (Figure 4D). Furthermore, the ROC values of the nomogram were 0.772 in training cohort (Figure 4E) and 0.629 in validation cohort (Figure 4F), indicating a satisfactory accuracy and reliability of the nomogram.

### 3.5 Functional characteristics of prognostic model

**FIGURE 5:**
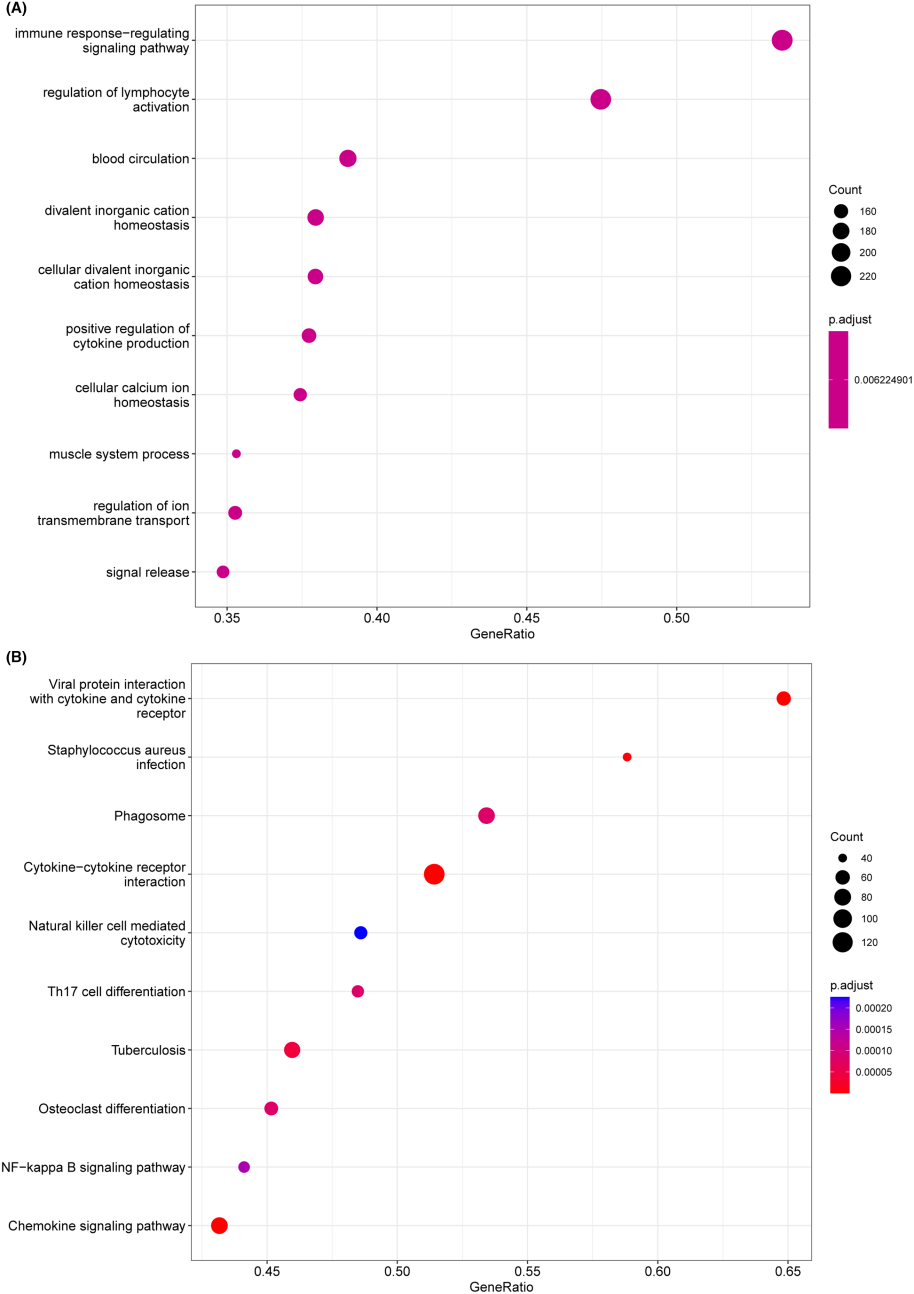
Functional enrichment analysis. (A) Gene ontology (GO) biological process (BP) analysis. (B) Kyoto Encyclopedia of Genes and Genomes (KEGG) analysis.

DGEs between high‐risk and low‐risk groups were identified with a threshold of |log_2_ (Fold Change)| >1 and *p* < 0.05. The GO enrichment analyses revealed that DEGs were significantly clustered in immune‐related pathways such as immune response‐regulating signalling and positive regulation of cytokine production pathways (Figure 5A). Moreover, the KEGG pathway enrichment analysis showed a significant enrichment in pathways of cytokine–cytokine receptor interaction pathway (Figure 5B). Taken together, these findings suggested that SASP‐related genes may play an important role in TME modulation and hence influence the prognosis of AML.

### 3.6 Characteristics of immune cells infiltration and therapy efficacy

**FIGURE 6:**
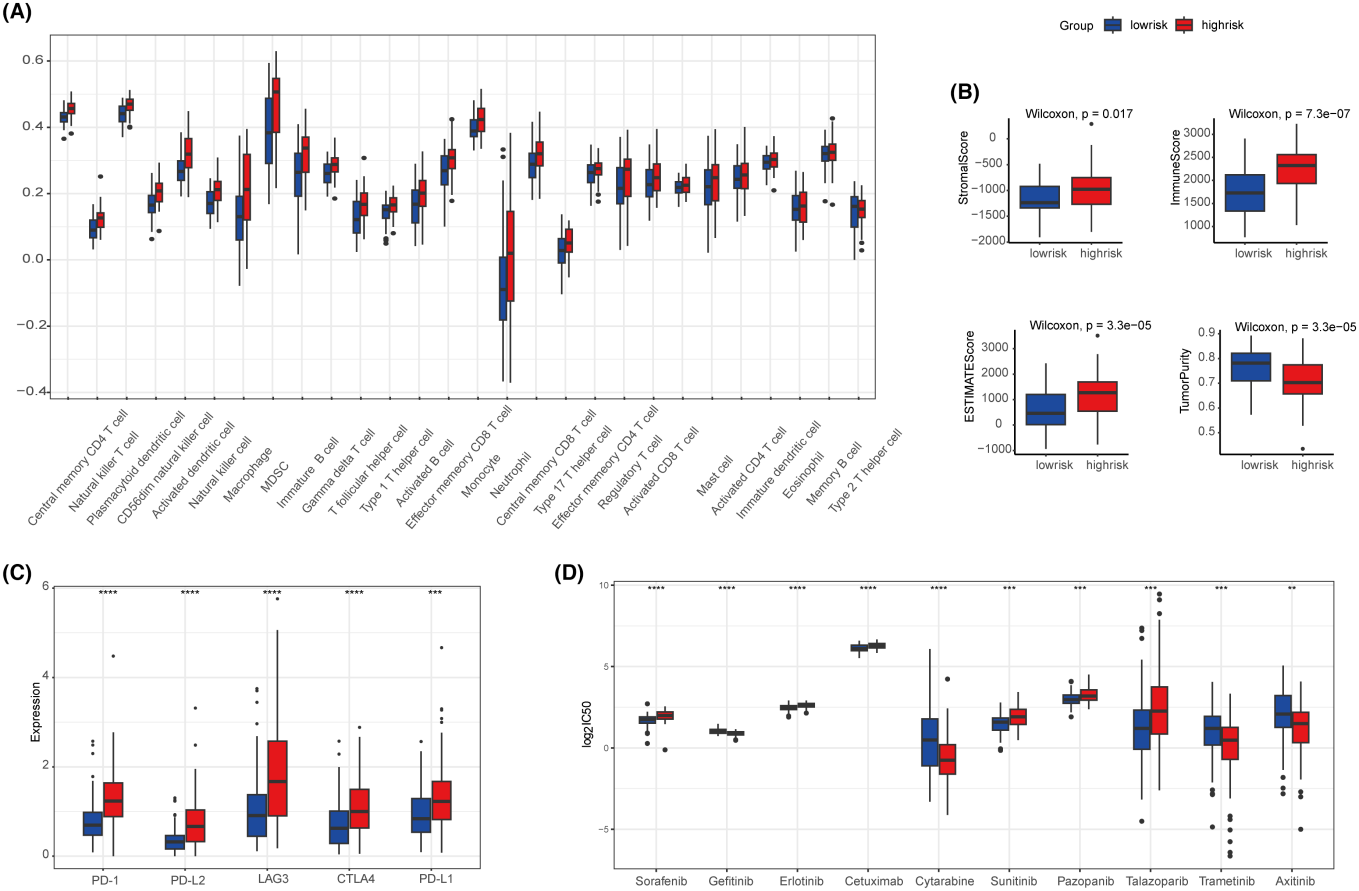
Immune cell infiltration and immunotherapy response of low‐ and high‐risk groups. (A) correlation of risk score and immune cell infiltration by ssGSEA. (B) RiskScore and ImmuneScore, StromalScore, ESTIMATEScore and TumorPurity correlation analysis. (C) Expression of *PD‐1*, *PD‐L2*, *LAG3*, *CTLA4* and *PD‐L1* in the two risk groups. (D) Chemotherapeutic Response in the two risk groups.

The results of ssGSEA showed that AML patients in high‐risk group have significant higher immune cell infiltrating level (Figure 6A). TME scores such as StromalScore, ImmuneScore and ESTIMATEScore were significantly elevated whereas TumorPurity was decreased in patients with higher risk score (Figure 6B). Furthermore, the expression of immune checkpoint genes including *PD‐1*, *LAG3*, *CTLA4*, *PD‐L1* and *PD‐L2* were significantly increased in high‐risk group (Figure 6C). Collectively, these results indicate that patients in high‐risk group are supposed to be more sensitive to immune checkpoint therapy. To further investigate the relationship between risk score and drug sensitivity, the IC_50_ of 251 chemotherapeutics were compared between high‐risk and low‐risk groups (Figure 6D). The results showed that patients in high‐risk group were more sensitive to 6 of top 10 significant chemotherapeutics (Sorafenib, Erlotinib, Cetuximab, Sunitinib, Pazopanib and Talazoparib) and patient in low‐risk group were more sensitive to the others (Gefitinib, Cytarabine, Trametinib and Axitinib), indicating the risk score might contribute to treatment planning.

## 4 DISCUSSION

AML is a highly aggressive and heterogeneous hematologic malignancy that affects the bone marrow and blood cells.^21^ Recent studies have demonstrated that SASP contributes to the development and progression of AML by creating a pro‐inflammatory environment in the bone marrow microenvironment. Understanding the role of SASP in AML may aid in the development of new therapies for this disease. In this study, we aimed to investigate the expression patterns of SASP‐related genes in AML, as well as their prognostic values and effects on the TME. Specifically, we constructed a prognostic signature with 11 differential expressed and prognosis associated SASP‐related genes including *G6PD, CDK4, RPS6KA1, UBC, H2BC12, KIR2DL4, HSF1, IFIT3, PIM1, RUNX3* and *TRIM21*. The AML patients were stratified into high‐ or low‐risk groups based on the median of risk score. Patients in high‐risk group suffered poor survival outcome in both of training and validation cohorts. Furthermore, multivariate Cox regression models revealed that the risk score was an independent risk factor for prognosis of AML.

In the present study, the 11 genes identified in the gene signature have been reported as prognostic biomarkers in various malignancies and play crucial roles in tumour formation. The acetylation regulation of *G6PD* is involved in the metabolic reprogramming of AML.^22^ The dysregulation of *CDK4* in multiple pathways leads to the uncontrolled growth of cancer cells.^23^ Up‐regulated expression of *RPS6KA1* results in chemoresistance and poor prognosis in patients with AML.^24^ *UBC* is a highly conserved polypeptide that is associated with a range of signal transduction and life processes.^25^ *KIR2DL4* can mediate a complicated cross‐talk between immune checkpoint and cytokines in breast cancer microenvironment and dictate distinct outcome of immunotherapy.^26^ *HSF1* is the transcriptional regulator of the heat‐shock response and various other cellular processes that are required for anabolic metabolism, cellular proliferation and tumorigenesis.^27^ The overexpression of *IFIT3* is connected to the chemotherapy resistance.^28^ *PIM1*, a serine/threonine kinase, may be involved in FLT3‐mediated leukemogenesis.^29^ *RUNX3* is an important regulator of human haematopoiesis, whose overexpression might contribute to the pathogenesis of AML.^30^ *TRIM21* plays an important role in proliferation and apoptosis of tumour cells and has been proposed as a potential target for therapy.^31^ These evidences strengthen the important roles of SASP‐related genes in tumour and their potential to predict the prognosis of AML.

The results of GO and KEGG enrichment analyses revealed that the differential expressed genes between the two groups mainly participated in immune‐related pathways, such as immune response‐regulating signalling pathway, positive regulation of cytokine production and cytokine‐cytokine receptor interaction, indicating that SASP might infect the outcome of AML patients via remodelling the tumour TME. Moreover, we explored the immune infiltration level and expression of immunotherapeutic targets in the two groups, and found that patients in the high‐risk group tend to be more sensitive to immunotherapies.

In summary, a prognostic signature for AML patients based on SASP was developed and validated. The nomogram that incorporated age, cytogenetic risk and risk score demonstrated a significant predictive ability for AML prognosis. Notably, the model was found to be closely associated with the immune microenvironment in AML through SASP, which could potentially serve as a target for therapy.

## AUTHOR CONTRIBUTIONS

**Ming‐Feng Li:** Data curation (equal); formal analysis (equal); visualization (equal); writing – original draft (equal). **Dong‐Hui Zhang:** Methodology (equal); resources (equal); validation (equal); writing – original draft (equal). **Li‐Si Wang:** Data curation (equal); formal analysis (equal); validation (equal); writing – original draft (equal). **Cai‐Feng Yue:** Methodology (equal); resources (equal); validation (equal); writing – review and editing (equal). **Li‐Juan Pang:** Conceptualization (equal); funding acquisition (equal); validation (equal); writing – review and editing (equal). **Yun‐Miao Guo:** Conceptualization (equal); funding acquisition (equal); resources (equal); writing – review and editing (equal). **Zhi‐Gang Yang:** Conceptualization (equal); funding acquisition (equal); supervision (equal); writing – review and editing (equal).

## FUNDING INFORMATION

This work was supported by the National Natural Science Foundation of China (82002866, 32100542, 82060054) and Guangdong Basic and Applied Basic Research Foundation (2023A1515030063, 2023A1515010212).

## CONFLICT OF INTEREST STATEMENT

The authors declare no competing financial interests.

## Data Availability

The datasets collected and analysed in the current study are available from the online repositories as described in the section of MATERIALS AND METHODS.
